# A First-In-Class, Humanized Antibody Targeting Alternatively Spliced Tissue Factor: Preclinical Evaluation in an Orthotopic Model of Pancreatic Ductal Adenocarcinoma

**DOI:** 10.3389/fonc.2021.691685

**Published:** 2021-07-29

**Authors:** Clayton S. Lewis, Aniruddha Karve, Kateryna Matiash, Timothy Stone, Jingxing Li, Jordon K. Wang, Henri H. Versteeg, Bruce J. Aronow, Syed A. Ahmad, Pankaj B. Desai, Vladimir Y. Bogdanov

**Affiliations:** ^1^Division of Hematology/Oncology, Department of Internal Medicine, University of Cincinnati College of Medicine, Cincinnati, OH, United States; ^2^Division of Pharmaceutical Sciences, University of Cincinnati College of Pharmacy, Cincinnati, OH, United States; ^3^Department of Environmental Health, University of Cincinnati College of Medicine, Cincinnati, OH, United States; ^4^Technology Development, LakePharma, Inc., Belmont, CA, United States; ^5^Einthoven Laboratory for Experimental Vascular Medicine, Department of Internal Medicine, Leiden University Medical Center, Leiden, Netherlands; ^6^Department of Biomedical Informatics, Cincinnati Children’s Hospital and Medical Center, Cincinnati, OH, United States; ^7^Division of Surgical Oncology, Department of Surgery, University of Cincinnati College of Medicine, Cincinnati, OH, United States

**Keywords:** pancreatic ductal adenocarcinoma, tissue factor, alternative splicing, monoclonal antibodies, orthotopic tumor model

## Abstract

In 2021, pancreatic ductal adenocarcinoma (PDAC) is the 3^rd^ leading cause of cancer deaths in the United States. This is largely due to a lack of symptoms and limited treatment options, which extend survival by only a few weeks. There is thus an urgent need to develop new therapies effective against PDAC. Previously, we have shown that the growth of PDAC cells is suppressed when they are co-implanted with RabMab1, a rabbit monoclonal antibody specific for human alternatively spliced tissue factor (asTF). Here, we report on humanization of RabMab1, evaluation of its binding characteristics, and assessment of its *in vivo* properties. hRabMab1 binds asTF with a K_D_ in the picomolar range; suppresses the migration of high-grade Pt45.P1 cells in Boyden chamber assays; has a long half-life in circulation (~ 5 weeks); and significantly slows the growth of pre-formed orthotopic Pt45.P1 tumors in athymic nude mice when administered intravenously. Immunohistochemical analysis of tumor tissue demonstrates the suppression of i) PDAC cell proliferation, ii) macrophage infiltration, and iii) neovascularization, whereas RNAseq analysis of tumor tissue reveals the suppression of pathways that promote cell division and focal adhesion. This is the first proof-of-concept study whereby a novel biologic targeting asTF has been investigated as a systemically administered single agent, with encouraging results. Given that hRabMab1 has a favorable PK profile and is able to suppress the growth of human PDAC cells *in vivo*, it comprises a promising candidate for further clinical development.

## Introduction

Pancreatic ductal adenocarcinoma (PDAC) is a highly lethal malignancy with most patients presenting at stage IV (1). Treatment of PDAC is hindered by its undruggable genomic drivers (mutated Kras, mutated p53), intra-tumoral heterogeneity, and desmoplasia that impedes drug entry into the tumor. The current chemotherapeutic standards of care, Gem-Abraxane and FOLFIRINOX, yield a median overall survival of only 9.6 months underscoring the need for more effective therapies (2).

Recently, we reported on the anti-tumor effects of RabMab1, our monoclonal antibody that binds the alternatively spliced isoform of human tissue factor (asTF), in a setting of orthotopic co-implantation with TF-expressing human PDAC cells (3). In humans and mice, the gene encoding tissue factor (*F3*) produces two protein isoforms: full-length TF (flTF), an integral transmembrane protein, and asTF, which has a unique C-terminus due to a single-nucleotide shift in its open reading frame. asTF lacks the alpha helical transmembrane domain present in flTF, rendering asTF a soluble secreted protein (4). flTF triggers blood coagulation by serving as an enzymatic cofactor for the serine protease fVII(a); flTF can also promote the activation of select protease-activated receptors (PARs) that control intracellular pathways governing platelet activation and wound healing (5). In contrast, asTF promotes cell proliferation, migration, and angiogenesis in a non-proteolytic manner by triggering the activation of PI3K/Akt, MAPK, and FAK pathways through its interactions with α6β1 and αvβ3 integrins (6). Elevated expression of total TF is positively associated with disease severity in several solid malignancies including breast, prostate, bone, and lower GI. One of the most extensively studied solid cancers with elevated TF expression comprises PDAC, where it was first identified as the protein responsible for PDAC cell-triggered coagulation (7). Elevated TF protein expression was first shown to correlate with the histological grade of PDAC in 1995 (8). Follow-up studies showed that TF promotes PDAC growth and invasion (9). TF localizes to the invasive front of PDAC and patients with higher TF levels in tumors have worse overall survival (10). Earlier studies of TF protein in human tissues were done using antibodies that could not discriminate between flTF and asTF; more recently, we reported that flTF and asTF are both overexpressed in breast cancer and PDAC (11, 12). In breast cancer, asTF fuels cancer cell growth *via* integrin ligation (13). This has also been seen in PDAC, where asTF promotes PDAC cell growth and spread in an orthotopic mouse model *via* β1 integrin-linked mechanisms (3).

Given TF’s ability to drive cancer cell growth and spread, therapeutic targeting of TF is an actively pursued area. Targeting flTF, however, is challenging due to an increased risk of bleeding and flTF’s expression in many normal tissues. In contrast to targeting flTF, targeting asTF – which is dispensable to normal hemostasis – is thus a potentially more attractive means to disrupt TF-mediated signaling; moreover, the expression of asTF in normal tissues is minimal compared to that of flTF (12). We have developed a rabbit monoclonal antibody termed RabMab1 that specifically recognizes an epitope unique to human asTF’s C-terminus. Tumor-suppressing properties of RabMab1 were evident in the breast cancer and PDAC settings showing that, when asTF-producing cancer cells were exposed to RabMab1, the activation of FAK, Akt, and MAPK pathways was suppressed *in vitro* and co-implanting RabMab1 with tumor cells suppressed their growth *in vivo* (3, 11). In this report, we describe RabMab1’s humanization and demonstrate that its humanized variant, termed hRabMab1, has an *in vivo* half-life well in the range of biologics currently used in the clinic. When administered intravenously in athymic nude mice, hRabMab1 is effective at stemming the growth of pre-formed orthotopic PDAC tumors.

## Materials and Methods

### Humanization, Construction, Expression, and Purification of hRabMab1

Antibody design was accomplished at LakePharma by generating a homology modeled antibody 3D structure and creating a profile of the parental antibody based on structure modeling. Acceptor frameworks to utilize were identified based on the overall sequence identity across the framework, matching interface position, similarly classed CDR canonical positions, and presence of N-glycosylation sites that would have to be removed. Two light chain (LC) frameworks IGKV1-39*01 and IGKV1-25*01 and two heavy chain (HC) frameworks IGHV3-23*01 and IGHV3-23*04 were selected for the humanization design. Humanized antibodies were designed by creating multiple hybrid sequences that fuse select parts of the parental antibody sequence with the human framework sequences. Using the 3D model, these humanized sequences were methodically analyzed by eye and computer modeling to isolate the sequences that would most likely retain antigen binding. The goal was to maximize the amount of human sequence in the final humanized antibodies while retaining the original antibody specificity. Back mutations were introduced and a total of three humanized light chains and three humanized heavy chains were designed. Humanness scores, representing how human-like the antibody variable region sequence is, were calculated according to Gao et al. (14). Briefly, an in-depth analysis of the humanness of therapeutic antibodies allowed the creation of a database of human antibody sequences, and increased humanness score was found to be correlated with decreased immunogenicity. Based on this method, heavy chain framework scores of 84 or above were considered human-like; light chain framework scores of 90 or above were considered human-like.

Full-length antibody genes for nine heavy/light chain pairs, as well as those encoding the rabbit chimeric parental, were codon-optimized, synthesized, and cloned into LakePharma’s proprietary high expression mammalian vector system and co-transfection of heavy and light chain plasmids at a 1:1 ratio was performed in HEK293 cells (Thermo Fisher Scientific) at 50 mL scale. Starting at 20 hours, and throughout the transient transfection production, antibody titers were measured (Octet QKe, ForteBio). Cultures were harvested at day 5 and antibodies in the conditioned media were purified using MabSelect SuRe Protein A resin (GE Healthcare).

### Assessment of asTF Binding by ELISA

96-well ELISA plates were coated with 5 ug/mL recombinant asTF in 0.05 M sodium bicarbonate buffer, pH 9.6. Serial dilutions of test antibody preparations were added to the pre-blocked plate and incubated for 1.5 hours at room temperature. The plate was then thoroughly washed and HRP conjugated secondary antibodies (Jackson) were added.

### Biosensor Affinity Testing

Multi-concentration kinetic experiments were performed on the Octet Red96 system (ForteBio). Anti-huIgG Fc biosensors (ForteBio 18-5064) were hydrated in sample diluent (0.1% BSA in PBS and 0.02% Tween 20) and preconditioned in pH 1.7 glycine. Antibody was diluted to 10 µg/mL with sample diluent and immobilized onto Anti-huIgG Fc biosensors for 120 seconds. After baselines were established for 60 seconds in sample diluent, the biosensors were moved to wells containing the antigen at a series of 7 concentrations, 3-fold serial dilution starting at 300 nM, to measure the association. Association was observed for 90 seconds and dissociation was observed for 90 seconds for each protein of interest in the sample diluent. The binding affinities were assessed by selecting the top 3 or 4 best-fit concentrations with a monovalent binding model (1:1 binding).

### Western Blotting

Human PDAC cell lines Pt45.P1 [a kind gift of Prof. Holger Katlthoff (12, 15)], PaCa44 [a kind gift of Prof. Stephan Haas (15)], AsPC1 and MIA-PaCa2 (both – ATCC) were utilized for antibody testing studies. Lysates were prepared with RIPA buffer and loaded into 10% TGX gels (BioRad). Protein was transferred onto PVDF membranes. Blocking was carried out using 2% BSA. RabMab1 and its derivatives were diluted to 1 µg/mL. Silver staining of 10% TGX gels was carried out using ProteoSilver Silver stain kit (Sigma PROTSIL1) according to the manufacturer’s instructions. All anti-asTF antibodies tested displayed high signal specificity; a protein species with a higher apparent molecular weight (~60 kDa) was seen in some lysate preparations (not shown).

### Cytotoxicity Studies

Antibody-dependent cellular cytotoxicity (ADCC) and cell-mediated cytotoxicity (CMC) assays were carried out using the aCella-TOX bioluminescence cytotoxicity kit (Cell Technology, Hayward, CA) according to the manufacturer’s instructions. For ADCC studies, NK cells were purchased from Cell Technology; for CMC studies, lyophilized sera (rabbit and human complement) was purchased from Sigma.

### qRT-PCR

RNA was isolated from cell lines and tumor tissue using the RNeasy kit (Qiagen). cDNA was prepared using Transcriptor Reverse Transcriptase (Roche). The primer/probe sets used for mRNA expression analysis are listed in the **Supplementary Material**.

### Migration Assay

Pt45.P1 cells were suspended in serum free DMEM and pretreated with the indicated antibody at a concentration of 50 µg/mL for 30 minutes. 1.5 x 10^4^ cells were then added to serum free DMEM in 8.0 µm transwells (Corning 353097) precoated with 1 µg laminin (Sigma L4544). 2% FBS in DMEM was used as the source of chemoattractants. ChromPure rabbit IgG (Jackson ImmunoResearch 011-000-003) was used as an isotype control.

### Animal Studies

University of Cincinnati’s Institutional Animal Care and Use Committee approved all animal studies. Antibody co-implantation studies: Pt45.P1 cells were preincubated with the indicated antibody diluted in PBS to a concentration of 5 mg/mL for 30 minutes prior to surgery. Human IgG1 isotype control (BioXCell, BE0297) was utilized as a negative control. 1x10^6^ cells were implanted with antibody into the pancreata of athymic nude mice. Pre-formed orthotopic tumor studies: 1x10^6^ Pt45.P1 cells were implanted into the pancreata of athymic nude mice (*Fox1^nu/nu^*; Jackson 002019). Mice were randomized into control and treatment groups. Tumor growth was followed for 7 weeks.

### Pharmacokinetic (PK) Assessment

cRabMab-hIgG1 or hRabMab1 were administered intravenously (IV) *via* tail vein to C57Bl/6 mice (n=16 and n=24 mice, respectively). Whole blood was collected at various time points by cardiac puncture method into EDTA coated tubes and centrifuged at 2000 x g for 15 minutes. Plasma was harvested, centrifuged a second time at 2000 x g for 15 minutes, and then frozen at -80°C until samples at all timepoints had been collected. Plasma aliquots were thawed on ice and diluted in the range of 1:1000 to 1:100,000 with PBS depending upon the standard plasma concentration profile for the human IgG1 in mice. Antibody concentration was determined using a human IgG ELISA kit (Abcam 195215). cRabMab1-hIgG1 and hRabMab1 standards were prepared in C57Bl/6 mouse plasma using serial dilution and used to obtain a standard curve.

PK antibody profile (plasma concentrations *vs* time data) after the administration of a single dose were analyzed using Phoenix^®^ WinNonLin^®^ version 8.1 (Certara L.P. (Pharsight), St. Louis, MO). The data were fitted employing a multicompartment PK model based on the apparent biphasic plasma-concentration vs time profile. Accordingly, PK parameters such as distribution and elimination half-life, systemic clearance, volume of distribution, and exposure (measured as the overall area under the plasma concentration-time curve or AUC) were computed. The PK parameters derived from this analysis were then utilized to simulate the multiple-dose PK profile and to predict steady state concentrations of hRabMab1 that may be achieved with repeated dosing. As a part of the pharmacodynamic studies conducted to assess the anti-tumor effects of the hRabMab1 antibody, blood samples collected at the end of the study were also analyzed to determine hRabMab1 concentrations in the plasma and compare the predicted *vs* observed steady-state concentration.

### Pharmacodynamic (PD) Assessment

1 x 10^6^ Pt45.P1 cells were implanted into the pancreata of athymic nude mice (*Fox1^nu/nu^*; Jackson 002019) and given 10 days to engraft, following which IV administration of hRabMab1, hIgG isotype control, or vehicle began at 18 mg/kg every other day (total of 22 treatment cycles). Mice were then euthanized *via* cardiac puncture while sedated with isoflurane. Whole blood was collected and plasma specimens harvested as detailed above. Tumors were measured with calipers and sectioned into 2 halves, one of which was snap frozen in liquid nitrogen and the other fixed in PBS with 10% formalin. Antibody concentrations in plasma were measured as detailed above.

### Immunohistochemistry and Image Analysis

10% formalin fixed tissues were embedded in paraffin and sectioned into 5 μm sections. Sections were stained as previously detailed (3). Briefly, sections were deparaffinized and rehydrated into DAKO wash buffer (Agilent S3006). Antigen retrieval was accomplished using a citrate solution (Biogenex HK086-9K). Native peroxidase activity was squelched using 0.4% hydrogen peroxide. Blocking was carried out with a Power Block solution (Biogenex HK085-5K) and sections were incubated with the following antibodies at the indicated dilution: CD31 (Abcam ab28364, 1:100), CD206 (Abcam ab6493, 0.1 µg/mL), Ki67 (Millipore Sigma ab9260, 1:100), HRP-conjugated anti-human IgG (Jackson ImmunoResearch 109-035-088, 1:500), HBEGF (Cell Signaling 27450, 1:50) and STN1 (Sigma HPA037924, 2.5 µg/mL). An HRP-conjugated anti-rabbit polymer (Agilent/Dako K4010) and DAB+ reagent were used to visualize primary antibody binding for CD31, CD206, Ki67, HBEGF, and STN1. Sections were counterstained with hematoxylin. Staining patterns were evaluated using Olympus BX51 equipped with Olympus DP72 digital camera; representative images were captured and used for statistical analyses. Staining intensity and/or positive staining events were analyzed using ImageJ.

### Gene Expression Analysis

RNA was isolated from tumors representing the median tumor volumes from each experimental cohort. RNA library preparation, next generation sequencing, and sequence alignment were performed by the CCHMC DNA Sequencing and Genotyping Core, Cincinnati, OH. 300 ng of total RNA, determined by Invitrogen™ Qubit™ high-sensitivity spectro-fluorometry, was poly-A selected and reverse transcribed using Illumina’s TruSeq^®^ stranded mRNA library preparation kit. Each sample was fitted with one of 96 adapters containing a different 8-base molecular barcode for high-level multiplexing. After 15 cycles of PCR amplification, completed libraries were sequenced using Illumina NovaSeq™ 6000, generating 30 million high-quality, 150 base long paired-end reads per sample. A quality control check on the fastq files was performed using FastQC. Upon passing quality metrics, the reads were trimmed to remove adapters and low-quality reads using default parameters in Trimmomatic [Version 0.33] (16). Trimmed reads were then mapped to a reference genome using default parameters with strandness (RF for paired-end) option in Hisat2 [Version 2.0.5] (17). In the next step, transcript/gene abundance was determined using kallisto [Version 0.43.1] (18). We first created a transcriptome index in kallisto using Ensembl cDNA sequences for the reference genome. This index was then used to quantify transcript abundance in raw counts and transcript per million (TPM).

### Statistical Analysis

1-way ANOVA was used to determine statistical significance in **Figures 2B, C**, **4A, D–F**, **5B, C** and **Supplementary Figure 1** (GraphPad Prism v6.0). *p ≤ 0.05, **p ≤ 0.01, ***p ≤ 0.001.

## Results

### Generation of Rabbit-Human Chimeric RabMab1 Antibodies

In our previous studies, hybridoma-derived RabMab1 suppresses tumor cell migration *in vitro* and tumor growth *in vivo* when co-implanted with pancreatic and breast tumor cells (3, 13). To humanize RabMab1, we first sequenced the genomic region of the hybridoma clone encoding RabMab1. The heavy and light chain portions of the variable regions were cloned and inserted into vectors (fee for service, Abcam). These constructs were then introduced into modified HEK-293 cells to generate a recombinant prep of RabMab1 (rRabMab1). Following validation, rRabMab1 served as the reference point for the humanization of RabMab1, the first step of which was the creation of human-rabbit chimeric antibodies and the assessment of their binding kinetics.

**Figure 1:**
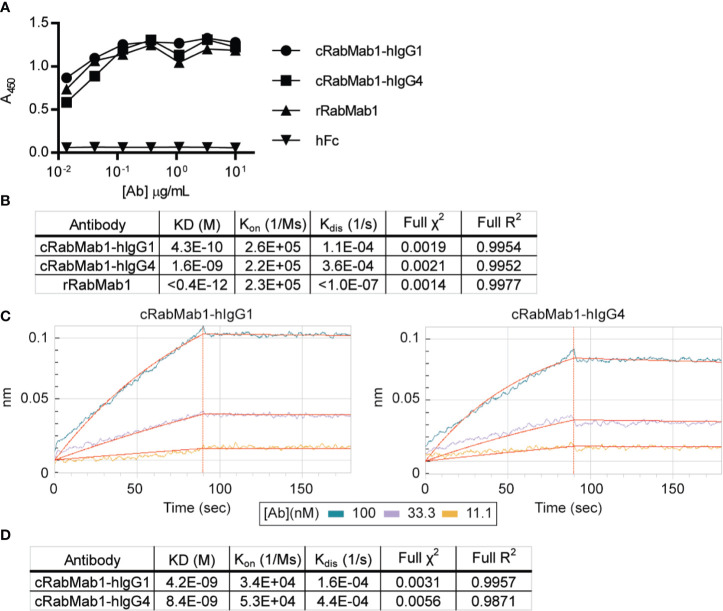
Chimeric RabMab1 antibodies have asTF binding affinities comparable to rRabMab1. Two human-rabbit chimeric RabMab1 antibodies were compared to parental rRabMab1 for their ability to bind asTF. **(A)** Serial dilutions of recombinant or chimeric RabMab1 were added to asTF-coated ELISA plates. Anti-human or anti-rabbit HRP conjugated secondary antibodies were used to assess antibody binding, hFc = non-targeting human Fc fragment. **(B)** HIS1K biosensor assessment of asTF binding kinetics of each antibody. **(C)** Trace results of AHC biosensor assessment of chimeric antibodies. **(D)** asTF binding kinetics as assessed by AHC biosensor experiments.

Chimeric antibodies comprised of the RabMab1 Fab region and a human Fc region characteristic of either an IgG1 or IgG4 isotype (herein, cRabMab1-hIgG1 and cRabMab1-hIgG4) were assessed by ELISA for their ability to bind recombinant asTF. The chimeras had comparable binding affinities compared to rRabMab1 (**Figure 1A**). The chimeric and parental RabMab1 antibodies were then further analyzed for asTF binding affinity using anti-penta-HIS (HIS1K) biosensors. cRabMab1-hIgG1 and cRabMab1-hIgG4 had asTF equilibrium dissociation constants (*K_D_*) of 430 pM and 1.64 nM, respectively; by comparison, rRabMab1 did not dissociate (**Figure 1B**, SPR sensograms can be found in **Supplementary Figure 1**). When assessed *via* anti-human IgG Fc capture (AHC) biosensors, cRabMab1-hIgG1 and cRabMab1-hIgG4 were found to have an asTF *K_D_* of 4.24 nM and 8.43 nM, respectively (**Figures 1C, D**). Given that hIgG1 isotype antibodies may activate the complement pathway and bind Fc receptors on phagocytic cells (19), as well as its lower *K_D_*, we elected to proceed with further development of cRabMab-hIgG1.

### cRabMab1-hIgG1 Binds Native Human asTF and Suppresses PDAC Cell Migration *In Vitro*

**Figure 2:**
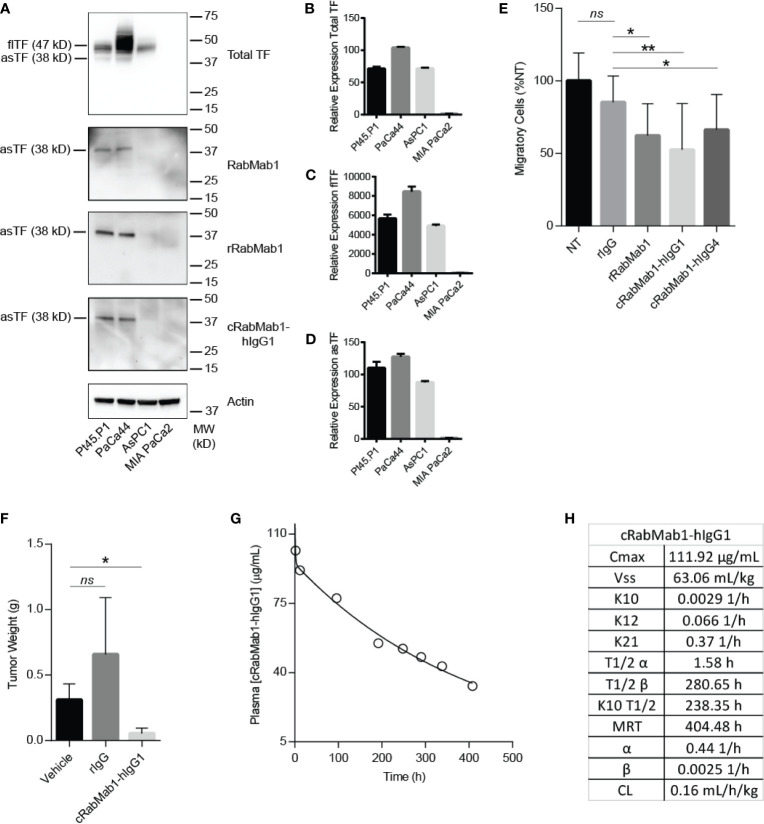
cRabMab1-hIgG1 suppresses tumor growth and has favorable pharmacokinetics. cRabMab1-hIgG1 was assessed for asTF binding *in vitro*, effects on tumor growth, and pharmacokinetics. **(A)** Western blot of Pt45.P1, PaCa44, AsPC1 and MIA-PaCa2 lysates (30 ug each). Each blot was probed with the indicated antibody at 1 µg/mL **(B)** qPCR for total TF transcripts showing relative expression levels. **(C)** qPCR for flTF transcripts showing relative expression levels. **(D)** qPCR for asTF transcripts showing relative expression levels. **(E)** Migration assay with Pt45.P1 cells treated as indicated. **(F)** Tumor weights - 1x10^6^ Pt45.P1 cells were incubated with either 100 µg of rIgG, 100 µg cRabMab1-hIgG1, or an equal volume of vehicle and implanted into the pancreata of athymic nude mice (n = 7/group); tumors grew for 45 days before sacrifice. **(G)** Plasma levels of cRabMab1-hIgG1 resulting from a single injection of 18 mg/kg of the antibody in C57Bl/6 mice. **(H)** PK analysis of cRabMab1-hIgG1. *p ≤ 0.05; **p ≤ 0.01; ns, not significant.

cRabMab-hIgG1 was next evaluated for its ability to bind asTF in immobilized, denatured, whole cell lysates of Pt45.P1, PaCa44, AsPC1 and MIA-PaCa2 cells: western blotting revealed that cRabMab-hIgG1 recognizes native asTF comparably to RabMab1 [**Figure 2A**; Total TF protein assessed using RabMab95 (20)]. qRT-PCR showed that TF isoform transcript levels for Pt45.P1, PaCa44, and MIA-PaCa2 corresponded to protein expression levels when normalized to TATA Binding Protein (**Figures 2B–D**); interestingly, while AsPC1 express asTF protein at very low levels, qRT-PCR revealed that asTF transcripts are relatively abundant in AsPC1 (**Figures 2A, D**). Our previous studies have shown that RabMab1 suppresses the migration of Pt45.P1 cells in a transwell migration assay (3). We, therefore, assessed the ability of cRabMab1-hIgG1 to inhibit PDAC cell migration in a transwell assay; cRabMab1-hIgG1 significantly suppressed the migration of Pt45.P1 cells (quantification is presented in **Figure 2E**, representative images of transwell inserts are shown in **Supplementary Figure 2**).

We next determined whether cRabMab1-hIgG1 can elicit CMC and/or ADCC. Pt45.P1 cells were pretreated with concentrations of cRabMab1-hIgG1 or human Fc fragment control ranging from 1 µg/mL – 1 mg/mL before being incubated with 25% human complement. Even at the highest concentrations of the antibody, only minimal lysis of the target cells was observed (**Supplementary Figure 3A**). Similarly, when Pt45.P1 cells were pretreated with cRabMab1-hIgG1 (concentrations ranging from 0.02 – 50 µg/mL) followed by 4 hours of incubation with Natural Killer (NK) cells (2:1 ratio NK : Pt45.P1), only minimal lysis of the target cells was observed (**Supplementary Figure 3B**).

### cRabMab1-hIgG1 Suppresses Tumor Growth *In Vivo*

Our previous studies have shown RabMab1 to suppress the growth of Pt45.P1 cells *in vivo* when co-implanted in an orthotopic setting (3). To determine whether this effect was preserved post-chimerization, we pre-treated Pt45.P1 cells with 5 mg/mL of cRabMab1-hIgG1 and then implanted 1x10^6^ cells in the pancreata of athymic nude mice. cRabMab1-hIgG1 reduced tumor growth by 78% compared to vehicle control, while no difference was observed between vehicle control and non-targeting antibody control (**Figure 2F**).

To evaluate the PK profile of cRabMab1-hIgG1, we injected it *via* tail vein into 16 C57Bl/6 mice at the dose of 6 mg/kg. Blood was collected at regular intervals for 17 days *via* cardiac puncture. As typically observed for monoclonal antibodies (21), cRabMab1-hIgG1 exhibited a biphasic decline with a short-lived distribution phase and an extended elimination phase. It had a distribution half-life of 1.6 hours and an elimination half-life of 281 hours. The steady state volume of distribution (V*ss*) was 63 mL/kg. The area under the concentration-time curve (AUC_0-t_) was observed to be 24.5 mg/mL/h (**Figures 2G, H**); please also see **Figure 2H** for additional PK findings.

### Humanization of cRabMab-hIgG1

**Figure 3:**
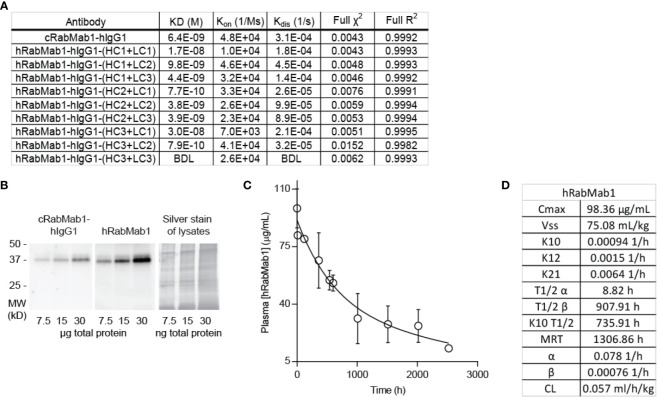
hRabMab1 binds asTF with high affinity and has a favorable PK profile. Nine fully humanized antibodies were produced through mutagenesis of the heavy chain and light chain encoding vectors of the variable region of rRabMab1; HC3+LC3 was chosen for further analysis. **(A)** asTF binding kinetics of humanized RabMab1 antibodies compared to cRabMab1-hIgG1 as determined by AHC biosensor analysis. BDL indicates values were below instrument detection limit. **(B)** Western blot of Pt45.P1 lysates comparing the asTF detection ability of cRabMab1-hIgG1 and hRabMab1 (each diluted to 1 µg/mL); Silver stain of lysates. **(C)** Plasma levels of hRabMab1 resulting from a single injection of the antibody in C57Bl/6 mice. **(D)** PK analysis of hRabMab1.

Given the encouraging results of our studies comparing cRabMab-hIgG1 and rRabMab1, we proceeded to humanize cRabMab-hIgG1. Plasmids encoding the heavy and light chains of the variable region of cRabMab-hIgG1 were mutagenized to achieve human characteristics. Multiple iterations of this process were performed leading to the creation of three heavy chain and three light chain variants. Combinations of each of these were used to create 9 humanized antibodies; the binding characteristics for each of these were assessed using AHC biosensors (**Figure 3A**, SPR sensograms can be found in **Supplementary Figure 4**). Using this readout, RabMab1-hIgG1-HC3+LC3, hereafter hRabMab1, displayed affinity in the picomolar range (beyond the instrument’s detection limit) due to an extremely slow off-rate. hRabMab1’s ability to detect asTF *via* western blot was the same as that of cRabMab1-hIgG1 (**Figure 3B**).

### hRabMab1 Suppresses PDAC Cell Growth *In Vivo*

Given the favorable *in vitro* results, we proceeded to test hRabMab1 *in vivo*. We first assessed the PK properties of hRabMab1 *via* IV administration of hRabMab1 (6 mg/kg) to C57Bl/6 mice. Plasma was collected from 2 mice per time-point as indicated. As with cRabMab1-hIgG1, a biphasic elimination curve was observed with distribution and elimination half-lives of 8.82 hours and 908 hours, respectively (**Figures 3C, D**). The steady state volume of distribution was 75.1 mL/kg. AUC_0-t_ value for hRabMab1 was observed to be 89.2 mg/mL/h; please also see **Figure 3D** for additional PK findings.

**Figure 4:**
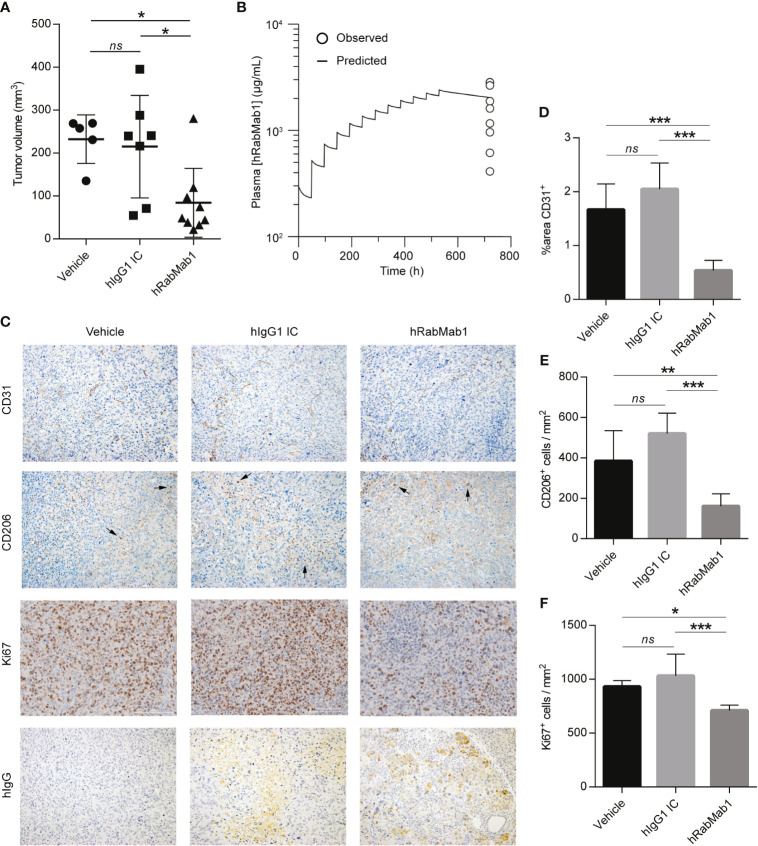
Systemic administration of hRabMab1 suppresses tumor growth. 1x10^6^ Pt45.P1 cells were orthotopically implanted into 21 athymic nude mice. Mice were treated every other day with vehicle, 18 mg/kg of hIgG1 IC, or 18 mg/kg of hRabMab1 for a total of 22 treatment cycles. **(A)** Tumor volumes. **(B)** WinNonLin prediction of hRabMab1 accumulation in plasma graphed alongside the amounts detected *via* hIgG1 ELISA at the time of sacrifice. **(C)** Representative images, immunohistochemical analysis of tumor sections as indicated; CD206 panel: arrowheads indicate positive signal (intensely stained single cells); original magnification: 20X. **(D)** Quantification of CD31 positivity in 9 representative images per treatment group. **(E)** The number of CD206^+^cells/mm^2^ in 9 representative images per treatment group. **(F)** The number of Ki67^+^cells/mm^2^ in 9 representative images per treatment group. *p ≤ 0.05; **p ≤ 0.01; ***p ≤ 0.001; ns, not significant.

We then proceeded with pharmacodynamic testing of hRabMab1. 1x10^6^ Pt45.P1 cells were implanted into the pancreata of athymic nude mice and given 10 days to engraft prior to the onset of treatment. Mice were then randomly divided into treatment groups receiving either hRabMab1, hIgG1 isotype control, or an equal volume of vehicle control *via* tail vein injection. Based on our previous co-implantation studies and hRabMab1’s PK parameters, we arrived at the treatment concentration of 18 mg/kg. hRabMab1 was administered every other day for a total of 22 treatment cycles. Mice treated with hRabMab1 bore tumors that were on average 1/3 the size of those treated with vehicle or isotype control (**Figure 4A**); no differences in average body weight and/or hematological profile were observed between the cohorts (data not shown). Analysis of plasma collected at sacrifice showed that mice treated with hRabMab1 had it circulating at a concentration that was consistent with what we predicted based on simulation of multiple dose administration using the PK parameters derived following single dose administration (**Figure 4B**).

Immunohistochemical analysis demonstrated a statistically significant ~70% reduction in CD31 staining in tumors treated with hRabMab1, pointing to a suppression of neovascularization (6); there was also a ~60% decrease in intratumoral M2-polarized macrophage accumulation (CD206) and a ~25% decrease in the number of actively proliferating cells (Ki67) in the hRabMab1 cohort when compared to the vehicle and/or isotype control cohorts (**Figures 4C–F**). When tumor specimens were stained with an anti-hIgG antibody, there was no signal in tumors treated with vehicle; a minimal, diffuse signal in tumors treated with isotype control; and a pronounced signal in tumors treated with hRabMab1, indicating its accrual in tumor tissue (**Figure 4C**).

### Downregulation of Genes Associated With Focal Adhesion, Cytoskeleton Maintenance, and Cell Cycle in hRabMab1-Treated Tumors

**Figure 5:**
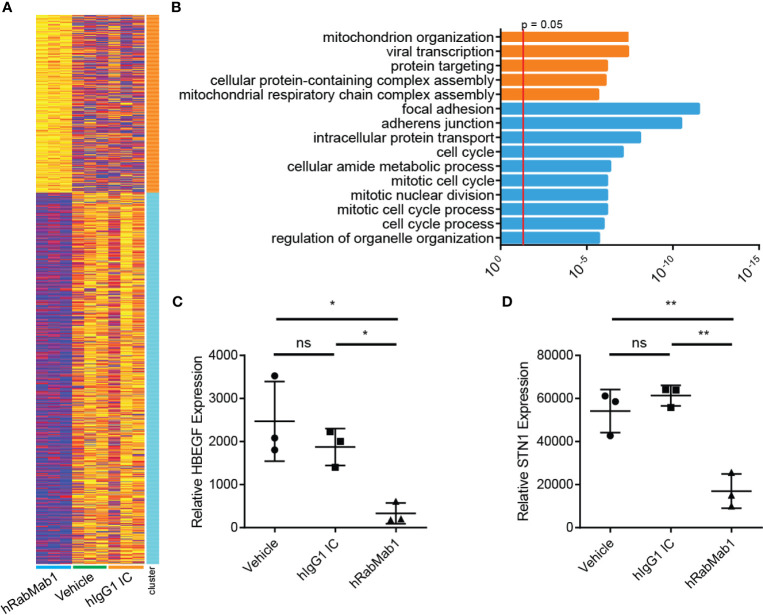
RNAseq analysis of tumor tissue harvested from pharmacodynamic studies. RNA was isolated from 3 tumors from each group representing the median tumors of that group. **(A)** Following library preparation clustering was performed using ToppGene from which a heat map was produced highlighting differences of mRNA expression levels between hRabMab1 treated tumors and those treated with either vehicle or IgG isotype control. **(B)** ToppGene functional enrichment analysis of RNAseq data differentially-expressed genes highlighting strong downregulation of gene transcripts associated with cell cycle and focal adhesion biological processes and cellular component when tumors are treated with hRabMab1. mRNA expression levels of **(C)** HBEGF and **(D)** STN1 in these tumors were quantified by qRT-PCR. *p ≤ 0.05; **p ≤ 0.01; ns, not significant.

**Figure 6:**
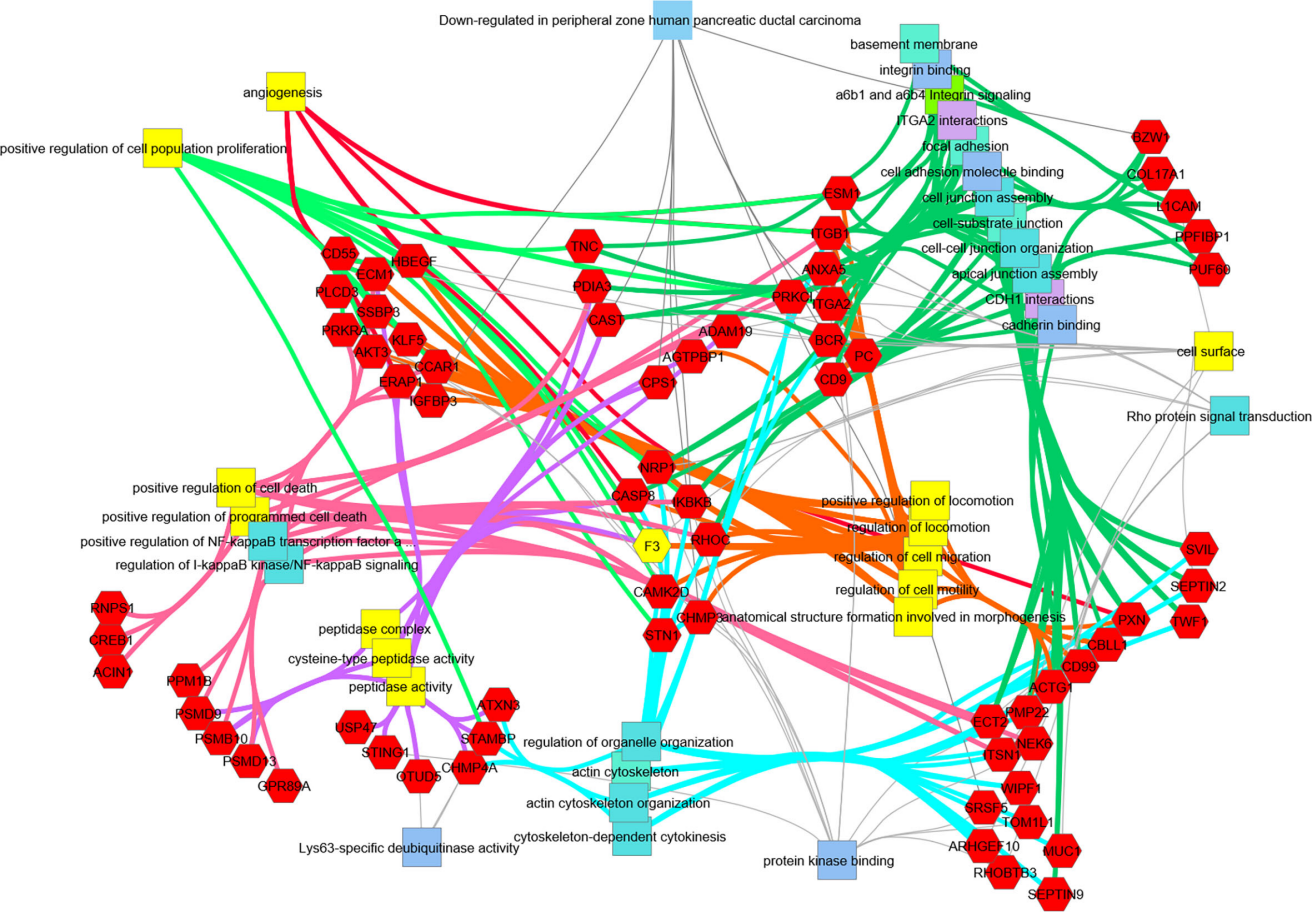
Transcripts downregulated in hRabMab1-treated tumors are associated with a large number of PDAC-relevant biological functions and pathways. Network analysis was performed using ToppCluster, which is based on ToppGene’s genelist functions and annotations including GO categories, reactome pathways, protein-protein interactions, and Pubmed manuscript associations. Categories known to be associated with *F3* (highlighted in yellow) also display a strong association with those gene function categories. Of particular interest, there is an extensive downregulation of focal adhesion, cell junction, and cell motility categories; in addition, downregulation of genes associated with positive regulation of proliferation is observed.

Differential gene expression analysis of hRabMab1-treated tumors revealed 955 ENSEMBL transcripts expressed at lower levels when compared to both vehicle and hIgG1 controls, and 458 that were expressed at higher levels (**Figure 5A**). Genelist enrichment analysis using ToppGene indicated that transcripts upregulated in the hRabMab1 group were most significantly associated with mitochondria. In contrast, transcripts that were downregulated in hRabMab1-treated tumors fell in several gene function categories including focal adhesion, cell motility, cell proliferation, cytoskeleton, regulatory proteases, and cell death, many of which are known be TF-associated (**Figures 5** and **6**). Among those most strongly downregulated were heparin binding epidermal growth factor (*HBEGF)*, CST complex subunit *STN1*, epithelial cell transforming 2 (*ECT2)*, phospholipase C delta 3 (*PLCD3)*, and ras homolog family member C (*RHOC)*. To validate our RNAseq findings, the expression levels of *HBEGF* and *STN1* were assessed by qRT-PCR (**Figures 5C, D**). These findings were confirmed on the protein level *via* IHC analysis (**Supplementary Figure 5**).

## Discussion

Because hybridoma-derived RabMab1 is effective at stemming tumor growth when co-implanted with cancer cells *in vivo* (3, 11), we pursued humanization of RabMab1. As we show here, target recognition was retained following the engraftment of the variable region of RabMab1 to a human constant region. When administered IV to mice, cRabMab1 had an extended elimination half-life, broad systemic exposure (peak plasma concentrations and AUC), and a volume of distribution suggestive of distribution to extravascular spaces. Humanization of the variable regions of RabMab1 yielded hRabMab1; this humanized antibody was verified to recognize native asTF. PK analysis showed that hRabMab1 had an elimination half-life of nearly 35 days in the circulation of C57Bl/6 mice. The observed persistence of high hRabMab1 levels in plasma, when viewed with the *in vitro* efficacy data, suggested that a multiple dosing regimen can be effectively employed to assess anti-tumor efficacy *in vivo*. As such, we implanted asTF-expressing human PDAC cells in the pancreata of athymic nude mice and allowed tumors to establish for 10 days before beginning IV treatment with hRabMab1. A significant reduction in tumor growth was observed, demonstrating hRabMab1’s efficacy as a single agent in mice. Consistent with our previous findings showing that asTF promotes neovascularization and macrophage infiltration by binding α6β1 and αvβ3 integrins expressed on endothelial cells, which triggers new vessel formation and, concomitantly, the expression of such cell adhesion molecules as E-selectin, VCAM1, and ICAM1 (3, 6, 22), we found that hRabMab1 suppresses neovascularization as well as macrophage infiltration in the tumor tissue. Blood samples collected to assay hRabMab1 levels at the end of the efficacy study provided a window of opportunity to compare the observed levels with those predicted by PK modelling. Although plasma samples were available only at one time point, the observed plasma concentrations were in good agreement with model-predicted values. This adds confidence that a PK-guided approach can be successfully employed to design efficacious dosing regimens for hRabMab1.

When it was discovered that TF is overexpressed in cancer tissues, systemically targeting it to control tumor growth and spread – as well as cancer-associated coagulopathies – became a goal of those working at the intersection of hematology and oncology. However, targeting “total TF” may result in bleeding due to a disruption of flTF’s function of maintaining the hemostatic envelope; we note that these issues may arise with the use of tisotumab vedotin, an “anti-total” TF antibody conjugated to a microtubule-disrupting agent (23). While the precise location of the epitope(s) recognized by tisotumab remain unclear, it does appear to bind flTF (24). Two milestone discoveries have changed the way we think of targeting TF. First, an antibody named 10H10 was invented (25) that binds flTF in a region that does not overlap with the binding sites of FVII and FX as indicated by resolution of the crystal structure of the Fab portion of 10H10 bound to the extracellular domain of flTF (26). Thus, 10H10 was believed to bind flTF in a manner that does not interfere with the formation of the extrinsic tenase complex, yet prevents the cleavage of PARs. This has recently come under scrutiny: new evidence showed that even though the 10H10 binding site on flTF does not overlap with the binding sites of FVII or FX, it could still disrupt this binding through steric interference (27); clinical trials have begun with 10H10 but its prospects are uncertain. Second, there was the discovery of asTF and the invention of RabMab1, an antibody that targets asTF’s unique C-terminus (4, 11). Given that asTF is dispensable for normal hemostasis, therapeutically targeting this TF isoform is likely to be less risky.

The use of monoclonal antibodies in the treatment of cancer has thus far yielded mixed results. While immunologically targeting the rogue immune cells of hematological malignancies has had great success, targeting solid tumors has only seen partial success, due in part to the inaccessibility of poorly vascularized portions of tumors. This is especially true of PDAC which is characterized by their poor vascularity and dense stroma. Despite this, according to clincaltrials.gov, >160 clinical trials featuring monoclonal antibodies have been launched targeting various aspects of PDAC biology. These can be broadly grouped into those that target glycosylated proteins of the cell-surface, growth factors, growth factor receptors, and checkpoint proteins. Anti-CEA and anti-mucin antibodies performed poorly in these trials; however, four active trials are currently being conducted with anti-CA19-9 antibody, MVT-5873. Anti-EGFR, anti-VEGF, and other antibodies aimed at growth factors and their receptors have also failed to elicit a response, likely owed to the fact that PDAC cells do not rely on any one particular growth factor pathway, but rather engage many such pathways, therefore inhibition of one is easily compensated for. A recent meta-analysis of 9 studies utilizing monoclonal antibodies that target growth factor pathways including cetuximab (anti-EGFR), bevacizumab (anti-VEGF), and ganitumab (anti-IGF1R) found there was no therapeutic benefit of these antibodies when combined with gemcitabine in the treatment of PDAC (28). Disruption of the tumor microenvironment with monoclonals directed against cell adhesion molecules and mucins has also largely been ineffective with the possible exception of anetumab ravtansine, a monoclonal antibody directed against mesothelin and conjugated with the maytansinoid DM4, an inhibitor of microtubule assembly, which is currently being assessed in a phase II trial as a part of a chemotherapeutic regimen (ClinicalTrials.gov Identifier: NCT03816358). The bulk of current clinical trials in PDAC utilizing monoclonal antibodies are focused on inhibiting the checkpoint pathway, despite the fact that when used as single agents, these have had little effect in the treatment of PDAC (29). There are, however, >100 clinical trials currently focused on assessing how these perform in combination with other chemotherapeutics.

As we show here, systemic administration of hRabMab1 as a single agent strongly suppressed tumor cell growth. RNAseq analysis of resected tumors pointed strongly to an inhibition of the expression of genes associated with focal adhesion and cell cycle progression in the hRabMab1 treated group. Given that the known route of asTF-mediated effects is through integrins, hRabMab1’s treatment effect on focal adhesion is to be expected and points to the existence of a positive feedback loop between asTF and integrin signaling. At the same time, downregulation of transcripts involved in cell cycle provides the framework for a mechanistic explanation of hRabMab1-mediated tumor growth inhibition. The fact that the cell cycle regulation genes most strongly suppressed by hRabMab1 are *HBEGF* and *STN1*, is novel and potentially significant because these genes’ protein products promote PDAC progression. Heparin-binding epidermal growth factor (HBEGF) is a member of the EGF growth factor family that binds EGFR along other ErbB receptors and acts as a mitogen (30). HBEGF and EGFR are both elevated in PDAC when compared to normal pancreas tissue (31). Hypoxia triggers the expression of *EGFR* through HIF2α in multiple cancer cell lines (32), and it was recently shown that *HBEGF* expression is activated in response to hypoxic conditions by HIF1α in the breast cancer cell line MDA-MB-231 (33). Interestingly, overexpression of asTF in Pt45.P1 cells promotes cell cycle progression and cell motility in hypoxic conditions through a β1 integrin-HIF1α-CAIX signaling cascade (34); thus, it is likely that neutralization of asTF by hRabMab1 mitigates signaling through this pathway leading to a suppression in *HBEGF* expression. Future studies will focus on how hRabMab1 affects the tumor microenvironment and intratumoral hypoxia. STN1 is one of the three components of the CST-complex, which helps maintain telomere integrity (35). The loss of STN1 dramatically impairs telomere replication, leading to rapid telomere shortening and the elicitation of DNA damage response (36). STN1 has also been identified as a mediator of chemotherapeutic resistance to DNA damaging agents; however, its suppression sensitizes cancer cells to these agents (37). In light of this finding, there is a strong rationale for combining hRabMab1 with gemcitabine or FOLFIRINOX in future pre-clinical studies.

In sum, we have shown that our first-in-class, humanized monoclonal antibody directed at asTF exhibits significant anti-PDAC tumor activity *in vivo* when administered intravenously as a single agent. Our findings strongly favor further development of hRabMab1 as a biologic for PDAC management.

## Data Availability Statement

The datasets presented in this study can be found in online repositories. The names of the repository/repositories and accession number(s) can be found below: https://www.ncbi.nlm.nih.gov/geo/, GSE158847.

## Ethics Statement

The animal study was reviewed and approved by IACUC, University of Cincinnati.

## Author Contributions

CSL, AK, KM, TS, JL, and JKW: carried out experiments, analyzed data, and contributed to writing of the manuscript. BJA and PBD: analyzed data and contributed to writing of the manuscript. SAA and HHV: contributed to writing of the manuscript. VYB: conceived the study and contributed to writing of the manuscript. All authors contributed to the article and approved the submitted version.

## Funding

This work was supported in part by grants R01CA190717 (NCI) and 17-65 BOGD (Pancreatic Cancer Action Network). CSL was supported by grant T32CA236764 (NCI).

## Conflict of Interest

Authors JL and JKW were employed by LakePharma, Inc.

The remaining authors declare that the research was conducted in the absence of any commercial or financial relationships that could be construed as a potential conflict of interest.

## Publisher’s Note

All claims expressed in this article are solely those of the authors and do not necessarily represent those of their affiliated organizations, or those of the publisher, the editors and the reviewers. Any product that may be evaluated in this article, or claim that may be made by its manufacturer, is not guaranteed or endorsed by the publisher.
