# Supplementary material for: A First-In-Class, Humanized Antibody Targeting Alternatively Spliced Tissue Factor: Preclinical Evaluation in an Orthotopic Model of Pancreatic Ductal Adenocarcinoma
Source: Front Oncol. 2021 Jul 29;11:691685. doi: 10.3389/fonc.2021.691685 (PMC8358774; doi:10.3389/fonc.2021.691685)
Supplement: Supplementary file 1 [file DataSheet_1.docx]

Supplementary Material

**Supplementary Methods**

| **Target** | **Forward Primer** | **Reverse Primer** | **Probe** |
| --- | --- | --- | --- |
| Total TF | 5'-CCAGAGTTCACACCTTACCTG | 5'-CAGGTGGGAACAAAAGTGAATG | 5'-/56-FAM/AGACAAACC/ZEN/TCGGACAGCCAACA/3IABkFQ/ |
| flTF | 5'-TGATGTGGATAAAGGAGAAAACTACTGT | 5'-CTACCGGGCTGTCTGTACTCTTC | 5'-/5TexRd-XN/TTCAAGCAGTGATTCCCTC/3IAbRQSp/ |
| asTF | 5'-GGGATGTTTTTGGCAAGGACTTA | 5'-CCAGGATGATGACAAGGATGATG | 5'-/56-FAM/AATCTTCAA/ZEN/GTTCAGGAAAGAAATATTCTACATCATTGGA/3IABkFQ/ |
| TATA Binding Protein | 5'-GTGGGGAGCTGTGATGTGAA | 5'-TGCTCTGACTTTAGCACCTGT | N/A |
| HBEGF | 5'-TTCTGGCTGCAGTTCTCTCG | 5'-AAGTCACGGACTTTCCGGTC | N/A |
| STN1 | 5'-TGCTTGAGCTGCCCACTATC | 5'-TCCGGTGGATCTTTCTGTGC | N/A |
| GAPDH | 5'-CCACCCATGGCAAATTCC | 5'-TCGCTCCTGGAAGATGGTG | 5'-/5Cy5/TGGCACCGT/TAO/CAAGGCTGAGAACGT/3IAbRQSp/ |

**Supplementary Table 1**: List of qRT-PCR primers and probes used in this manuscript

**
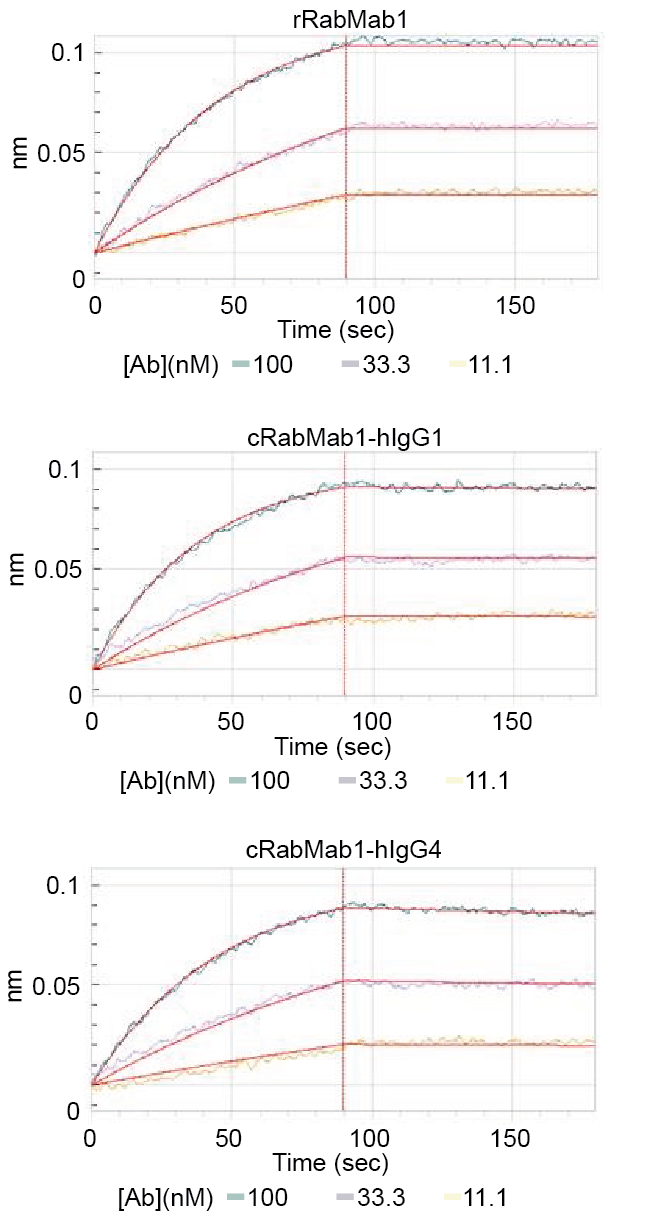
Supplementary Figures**

**Supplementary Figure 1.**  SPR sensograms for rRabMab1, cRabMab1-hIgG1, and cRabMab1-hIgG4 binding to asTF.**
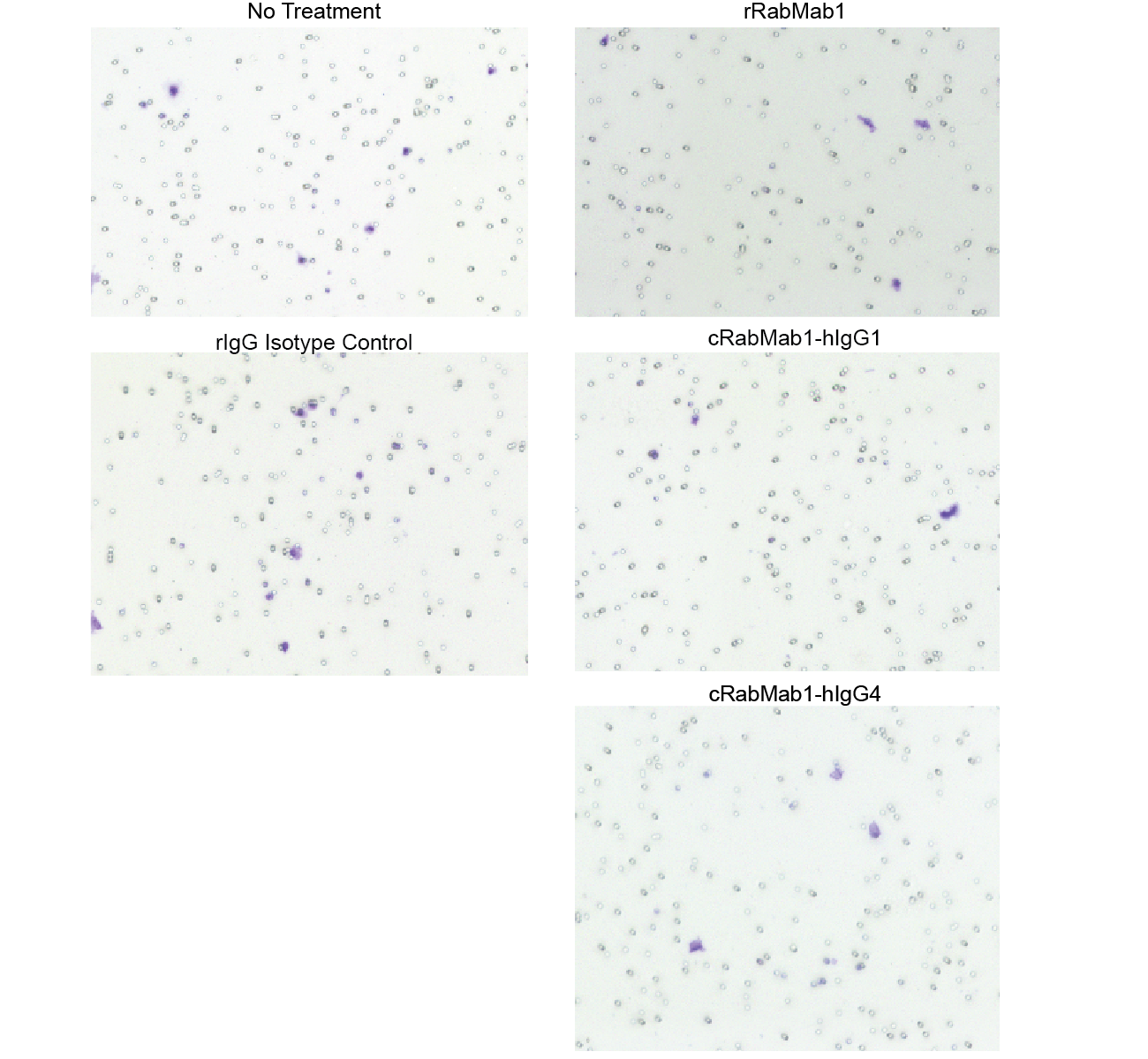
**

**Supplementary Figure 2.** Representative images, transwell migration assay. Pt45.P1 cells were pretreated with the indicated antibody at 50 µg/mL for 30 minutes prior to migration; 2% FBS was used as the source of chemoattractant. 15-25 fields were captured for each insert, data captured, and analyzed using GraphPad Prism.


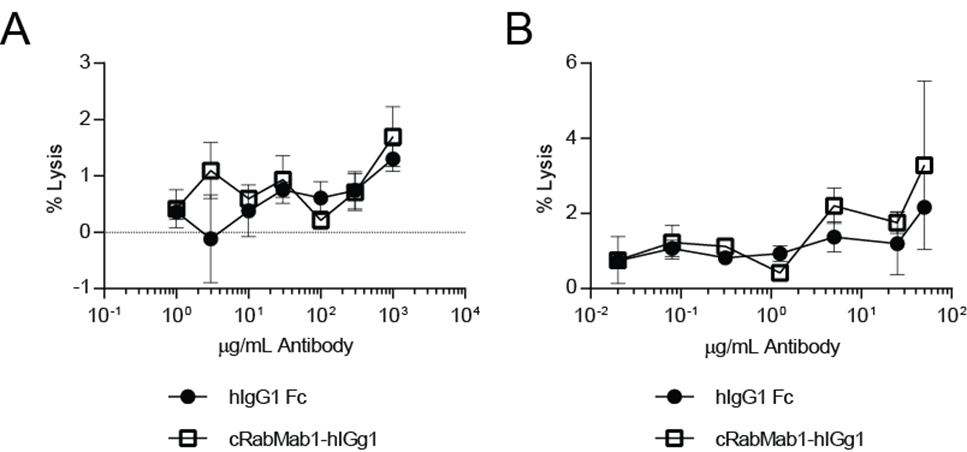


**Supplementary Figure 3.** cRabMab1 does not elicit a complement-mediated cytotoxic response (A) and/or cytotoxicity in response to the addition of NK cells to Pt45.P1 cells pretreated with cRabMab1 (B).


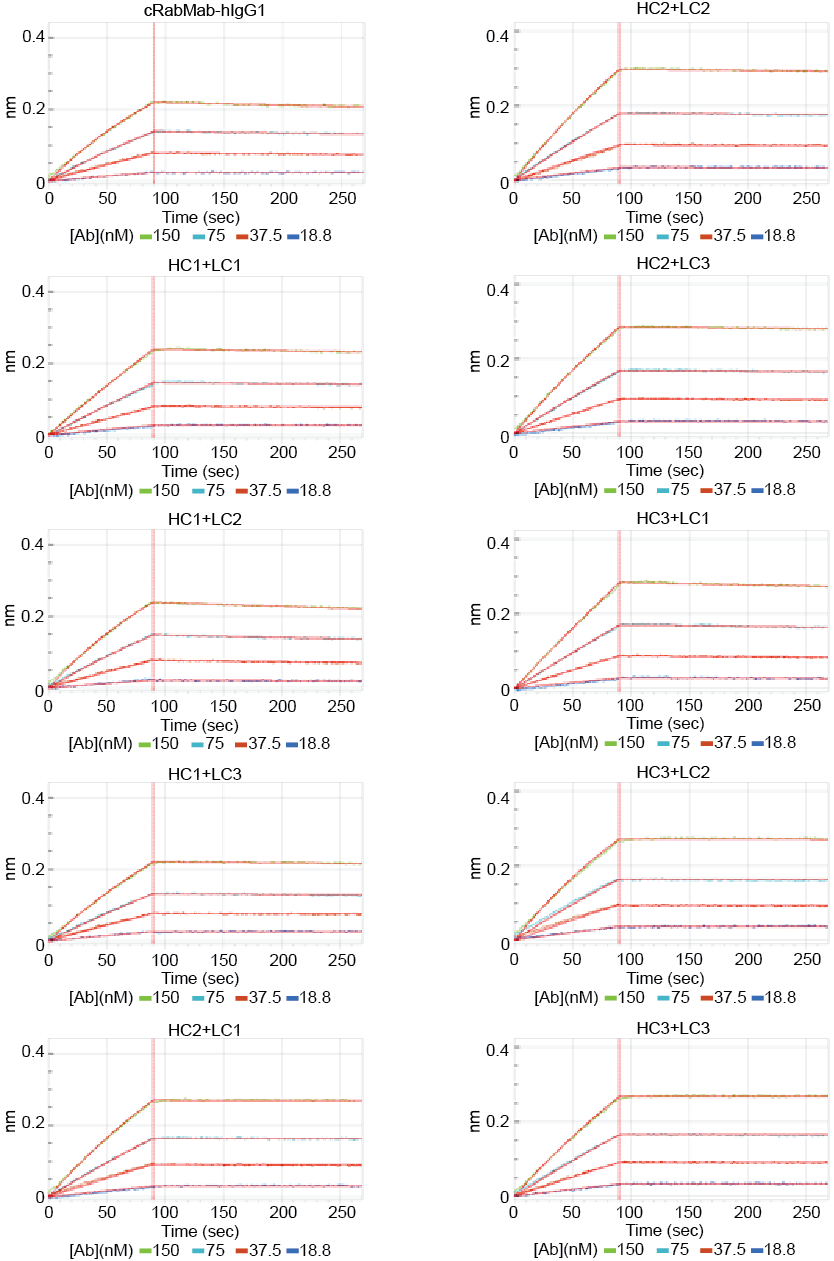


**Supplementary Figure 4.** SPR sensograms for binding of cRabMab1-hIgG1 and the nine humanized variants created by combinations of HC1, HC2, and HC3 with LC1, LC2, and LC3.


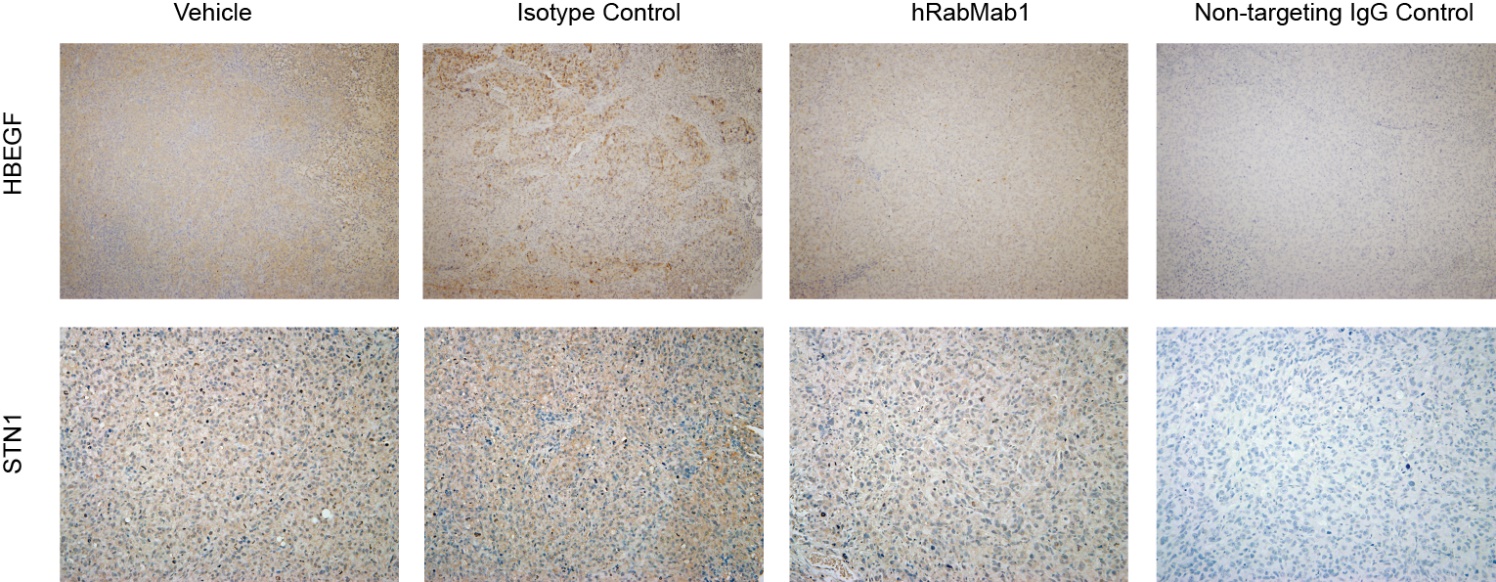


**Supplementary Figure 5**: Representative images, immunohistochemical analysis of tumor sections for the relative abundance of HBEGF and STN1 protein in the tumor tissue.
